# The role of social media during the COVID-19 pandemic: Salvaging its ‘power’ for positive social behaviour change in Africa

**DOI:** 10.34172/hpp.2022.03

**Published:** 2022-05-29

**Authors:** Roda Madziva, Brian Nachipo, Godfrey Musuka, Itai Chitungo, Grant Murewanhema, Bright Phiri, Tafadzwa Dzinamarira

**Affiliations:** ^1^School of Sociology and Social Policy, University of Nottingham, United Kingdom; ^2^Ministry of Health and Child Care, Harare, Zimbabwe; ^3^ICAP at Columbia University, Harare, Zimbabwe; ^4^Chemical Pathology Unit, Department of Laboratory Diagnostic and Investigative and Sciences, Faculty of Medicine and Health Sciences, University of Zimbabwe, Harare, Zimbabwe; ^5^Unit of Obstetrics and Gynaecology, Department of Primary Health Care Sciences, Faculty of Medicine and Health Sciences, University of Zimbabwe, Zimbabwe; ^6^ICAP at Columbia University, Pretoria, South Africa; ^7^School of Health Sciences & Public Health, University of Pretoria, Pretoria, 002, South Africa

**Keywords:** COVID-19, Social media, Public health, Communication

## Abstract

The ongoing coronavirus disease 2019 (COVID-19) pandemic remains a significant global public health crisis. The unique evolution of the COVID-19 pandemic has seen social media emerging and growing into an important vehicle for rapid information dissemination. This has in turn given rise to multiple sources of information, leading to what has come to be known as ‘infodemic’, associated with the plethora of misinformation and conspiracy theories. In this perspective, we explore the growth of the social media industry and the impact it has had during the ongoing COVID-19 crisis. We argue that while the multiple information pieces circulating on social media cause misinformation and panic, this might not necessarily and in all the cases influence sustained behaviours in the target population groups. We offer suggestions on how the power of social media can be harnessed and integrated into social and public health for a better digital balance for communication for development.

## Introduction

The severe acute respiratory syndrome coronavirus 2 (SARS-CoV-2), the aetiological virus for coronavirus disease 2019 (COVID-19), remains a significant global public health concern. Since the initial detection of the virus in China in late December 2019, the unfolding COVID-19 pandemic has seen social media growing as an important vehicle for rapid information dissemination. The imposition of lockdowns, need for physical distancing and other movement restrictions in many countries, as part of the COVID-19 response measures, resulted in growing numbers of people turning to social media in order to keep connected and informed about the pandemic.^1^ Thus, besides the COVID-19 pandemic proving to be the worst public health crisis of our time, it has also proved to have influenced the acceleration of communication technologies and digital platforms in the 21st century.^2^ COVID-19 has thus been dubbed ‘a digital pandemic’^3^ which has helped to create ‘internet citizens’ or ‘netizens’.^4^ In this perspective, we discuss the growth of the social media industry and its impact during the COVID-19 crisis and how during the same era, public health practitioners and communications experts can harness the power of social media for communication for development.

## Defining social media in the context of a public health global crisis

The term social media refers to computer/internet-based technology, and channels of mass-personal communication that facilitate interactions among users and through virtual networks and communities.^5^ In this way, social media make it easier for ordinary people to communicate widely, allowing anyone to broadcast information to a mass audience, and enabling the sharing of information, documents, videos, and photos. Some of the most popular social media websites as of January 2021 include Facebook (2.74 billion users); YouTube (2.29 billion users), WhatsApp (2 billion users), Instagram (1.22 billion users), TikTok (689 million users), QQ (617 million users), Douyin (600 million users) and Sino Weibo (511 million users).^6^

Amid the current state of the global COVID-19 pandemic and people’s desperation for information, social media, due to the communicative affordances they provide, including immediacy and wide dissemination, have provided the public with the democratic agency to generate conversations across diverse platforms. This has seen a dramatic increase in the proliferation of information and generating what has been termed ‘infodemic’, associated with various conspiracy theories about the pandemic.

An infodemic entails the circulation of an over-abundance of information including false or misleading information, leading to confusion, mistrust in health authorities and thereby undermining disease response, making it ‘hard for people to find trustworthy sources and reliable guidance when they need it’.^7,8^ In an information age, where communication hierarchies have been flattened, and the trust deficit between citizens and political leaders is widening, people increasingly struggle to make informed decisions, and often they trust the information they receive through their social media than what is disseminated via traditional mainstream media largely perceived as government controlled.^9^

## Misinformation and disinformation at global scale in a public health crisis

While the COVID-19 pandemic emerged as a global crisis that required immediate intervention, there was no clarity on the origin and causes of the virus during the initial days. Instead, this period was characterized by blame games between America and China, with the World Health Organization (WHO) inadvertently but equally embroiled in this diplomatic quagmire. This provided a conducive environment for social media users to fill this information vacuum. Unfortunately, this void was filled with an abundance of either misinformation or disinformation, which posed serious challenges in relation to ‘knowledge legitimacy, epistemic authority and competing truth claims’.^10^ Misinformation relates to false information that is circulated without the “disseminators’ knowledge” while disinformation refers to “all forms of false, inaccurate, or misleading information designed, presented and promoted to intentionally cause public harm or for profit”.^11^

Take for instance the former USA President Donald J. Trump’s use of the ‘#Chinese virus” hashtag on March 16, 2020 as a typical example of disinformation. This was not only noted to be misleading but was further attributed to the rise of Anti-Asian hashtags on Twitter and the subsequent fueling of xenophobic and anti-Asian sentiments worldwide.^12^ Such disinformation compelled WHO to warn against the use of terminology that connects diseases to countries or specific groups of people, and rather recommended the use of the WHO’s official name for the disease #covid19.

Social media conversations around the COVID-19 pandemic have also helped to propagate various conspiracy theories, including those linking the pandemic to the development of the 5G technology through the use of hashtags such as #5GCoronavirus. These hashtags gained traction on such social media platforms such as Twitter and Instagram.^13^ Another strand of conspiracy theories related to the Microsoft co-founder and philanthropist, Bill Gates who has been alleged to have created the coronavirus with the intention to control the global population and assume control of the global health system.^14,15^ Several posts were shared on Facebook, Twitter and YouTube, accusing Gates of using the COVID-19 pandemic as a pretext for mandatory vaccination.^16^ Another claim was that vaccines are only a cover for implanting some form of microchip, considered by especially Christian clergymen to be the Satanist ‘Mark of the Beast’.^17,18^ The few cited examples above further compounded public mistrust, skepticism and resistance to official information on COVID-19; thus, complicating the implementation of public health responses as citizens increasingly made use of the conspiracy theories as counter weapons to challenge official narratives about the pandemic.

Given that social media has enabled the creation of an information ecosystem that is not only swift, but also increasingly global, cross-pollination of conspiratorial scripts across geographical and national boundaries has become the order of the day.^19^ In particular, the Gates accusations while they have had worldwide impact especially in terms of reinforcing public vaccine hesitancy, the impact has been considerably noticeable in Africa where preexisting conspiracy theories around Gates’ neocolonial effort to suppress African births through vaccination were still in circulation,^20^ especially now exacerbated on social media.

In Africa, the COVID-19 pandemic created a conducive environment for old conspiracy theories to be resuscitated and for new ones to emerge. A study by Mutanga, Ureke, and Chani which used hashtags #COVID19SA, #CoronaVirusSA, #CoronaVirusZW, #Covid19Zim to evaluate the impact of social media in propagating the COVID-19 pandemic in South Africa and Zimbabwe has shown that international conspiracy scripts have increasingly been localized.^4^ The study reveals that the words that featured prominently in tweets include “G5, “Vaccine”, “Africa”, “Bill Gates”, “Trust” and “microchip”. This is one example which helps to demonstrate how localised and globalised conspiracy narratives can be mutually reinforcing.^4,19^ The same study ^4^ further shows that one of the events that generated a lot of interest on Twitter at the time was the pronouncement on television by French scientists Jean-Paul Mira and Camille Locht that the vaccine trials should be conducted on Africans. This saw a proliferation of comments on social media, especially from people of an African origin expressing their disappointment over the infamous proposal. This included the prominent African footballers Didier Drogba’s (Ivory Coast) famous tweet: “Africa isn’t a testing lab” which has been retweeted on several platforms using hashtags such as #africansarenotlabrats; #africansarenotguineapigs. This also became muddled up with the pre-existing anti-vaccine and anti-Gates theories thereby contributing to increased vaccine hesitancy.^21^

## Limitations of social media

### The misnomer of a magic bullet effect

There is a wide perception that messages shared on social media have a ‘magic bullet’ effect on the communities or audiences that receive those messages. This perception has been largely discredited by academics because of its suggestion that all members of an audience interpret messages in the same way and are largely passive receptors of messages. This theory does not consider intervening cultural and demographic variables such as age, ethnicity, gender, personality, or education that cause us to react differently to the media messages we encounter.^22,23^ Whilst some may be swayed by the messages on social media, it is important to note that people have their own pre-conceived judgements and perceptions that shape how they receive and decode messages shared on social media or through any other channel.

### Behaviour Change is a process

It does not always pan out that as people are exposed to messages on social media, they immediately are influenced to behave or act in a particular way. Behaviour change is generally not an event, but a process. The Transtheoretical Model or stages of change theory construes change as a process involving progress through a series of five stages. The model involves emotions, cognitions, and behavior.^24^ People at different touch points along this continuum have different informational needs, and benefit from interventions designed for their stage.^25^ The five stages are pre-contemplation; contemplation; preparation; action; and maintenance. In this regard, social media messages might not necessarily always result in specific behaviour change, especially if not done systematically. The experience is that social media messages during the pandemic have generally been once off messages without a specific build into a clear objective direction.

## Salvaging the infodemic into communication for development

Whilst social media arguably has had a “powerful” impact on creating and influencing perspectives regarding COVID-19, it should be noted that there are some potentially significant limitations to its impact and effects. Undoubtedly, social media contributes to the spread of information at a fast pace across the globe and different communities. However, this information does not always translate to knowledge and most importantly behaviour change, particularly if not conducted in a scientific way and systematic fashion. Information alone is insufficient to support behaviour change.^26^ In this regard, the multiple information pieces circulating on social media during the pandemic, cause misinformation and panic, but might not necessarily and in all the cases influence sustained behaviours in the target population groups. This gives rise to the opportunity for public health promoters to find the niche, the right space for deliberately exploring the strategic use of social media for reinforcing public health messages that would influence positive behaviour change outcomes in the midst of the COVID-19 infodemic.

Social media goes beyond just being a channel or tool of communication. Social media is about conversations, community, connecting with the audience and building relationships. It is not just a broadcast channel or a sales and marketing tool. Therefore, social media can actively be used as part of communication for development. Communication for Development (C4D) is about unlocking the solutions that will help people create change - for themselves, their families, their communities, or for a social cause.^27,28^

In this context of COVID-19, whilst it is noted that social media has done a lot of harm, there is hope and potential of using social media in a more systematic and deliberate manner for the purposes of influencing positive behaviours in specific communities. Leveraging social media for social and behaviour change communications is a natural and needed next step for addressing large scale challenges, including the ones presented by COVID-19.^29,30^ An example is that of the low-touch social media campaign produced by Population Foundation of India and delivered through Facebook Messenger was effective in promoting online information-seeking behaviors and reshaping gender attitudes of social media users.^31^ Below are some potential steps in rising above the infodemic and effecting positive behaviour change through social media

### Co-ordinated human centered design campaigns

Embarking on a specific social media campaign during the pandemic can potentially bear much more positive results in reaching out to key audiences. Unlike the uncoordinated messages going viral on social media, developing a campaign with specific objectives that are developed and delivered through human centred design approaches can bear positive results that influence behaviour change. Human centered design is an approach to creating solutions for problems and opportunities through a focus on the needs, contexts, behaviors, and emotions of the people that the solutions will serve.^32,33^

### Audience segmentation

Audience segmentation is important part of human centered design approach to developing a successful campaign. Most information being literally thrown on the social media is not carefully curated to the needs, desires and aspirations of specific target audience. Amidst the ‘noise’ on social media on COVID-19 a counter approach can be employed with a clear specific target group. For instance, whilst some have been swayed against COVID-19 vaccines through social media, embarking on a formal campaign via social media, targeting specific gatekeepers who access social media can help change their believes, attitudes and perceptions towards vaccines and in turn their influence can positively affect their communities, through other channels. Social media offers unique opportunity for targeting capabilities that allow identifying potential beneficiaries or audiences at a very granular and detailed level. This lets social media campaigns to be designed with the richness of people’s different profiles in mind, and gain power and effectiveness as a result.^34,35^ Audience segmentation will allow for the exploration and deeper understanding of key audiences and what their barriers to vaccination are and motivators and what is needed to make them advocates for vaccination.

### Journey maps

Every individual and community go through their own journey with regards to beliefs, attitudes and access to health products and services. In this regard, using human centered design approaches, one can be able to develop appropriate messaging and interventions delivered via social media and structured to provide appropriate information that supports their journey to vaccination, for instance. The intervention support individuals’ journey will help push individuals from e.g. precontemplation stage to contemplation and finally action as espoused in the Transtheoretical model.^24^ This goes in line with the WHO recommendation on infodemic management that aim to enable good health practices through 4 types of activities: listening to community concerns and questions; promoting understanding of risk and health expert advice; building resilience to misinformation and engaging and empowering communities to take positive action.^36,37^

### Locally contextual vantage points for beating the infodemic

For resource limited settings like Zimbabwe where access to social media is potentially high, but sustained use is limited especially among young people due to data related high costs, there is need to consider most sustainable approaches. Heavy flow of social media messages (audio, high resolution videos and images) potentially become expensive as well as compromising the storage space on cost sensitive smart phones that they use. In this regard, public health specialist can explore the use of offline interventions that allow for once off download of the app and the audience can continue to enjoy accessing the content and message even without data in their phones. This strategy capitalizes and establishes trustworthy sources of messages on COVID-19 and vaccines. “Social media campaigns can promote the download and sustained use of development apps, complementing offline interventions.^35^

### Behavioural nudges

As the infodemic continues to wreak havoc, there are some positive behaviours that can potentially be picked up by the audiences sub-consciously. One key innovation that has been discovered in the scientific study of behaviour change is that many factors can significantly impact people’s behaviour, yet bypass their conscious decision-making, attitudes, goals and awareness. An example. Being behavioural nudges.^38^ Behavioural nudges have been formally used in different settings to influence certain behaviours. Behavioural nudges are environmental cues that signal a desired response from the end user or channel their decision making, for example placing fruit at eye level to encourage consumption.^38^ Although not tested, there is potential of the many videos and memes circulating on social media to reinforce positive behaviours such as the wearing of masks. A video of an antivaxxer on social media speaking against vaccines but at the same time talking whilst wearing a facemask can act as a behavioural nudge on the importance of wearing a facemasks. Either way, some level of communication can be happening, intentional or not.

## Conclusion

The dangers of infodemic due to social media during the COVID-19 pandemic cannot be overemphasized. However, we also cannot ‘throw this powerful tool’ away because of the misuses and sometimes panic that it has caused. There lie opportunities to harness the power of social media and employ the scientific and artistic approaches of Human centered design to convey positive behavior change messages and improve the quality of life of the audiences via social media. By partnering with the fields of behavioral science and impact evaluation, as well as researchers, technical specialists, and social media experts, we can maximize these initiatives’ effectiveness and inclusiveness, and get a better sense of their impact during a pandemic.

## Authors’ contributions

RN, BN, GM (Godfrey Musuka) and TD prepared the first draft of the paper and GM (Grant Murewanhema) critically reviewed it. Subsequent reviews and revisions were completed by all authors. All authors reviewed the final draft and gave approval to publish.

## Funding

This research received no external funding.

## Ethical approval

Not applicable.

## Competing interests

The authors declare that they have no competing interests.
